# Drug Susceptibility Profiling of *Prototheca* Species Isolated from Cases of Human Protothecosis

**DOI:** 10.1128/aac.01627-22

**Published:** 2023-03-21

**Authors:** Angelika Proskurnicka, Kinga Żupnik, Zofia Bakuła, Mateusz Iskra, Uwe Rösler, Tomasz Jagielski

**Affiliations:** a Department of Medical Microbiology, Institute of Microbiology, Faculty of Biology, University of Warsaw, Warsaw, Poland; b Institute for Animal Hygiene and Environmental Health, Freie Universität Berlin, Berlin, Germany

**Keywords:** *Prototheca* species, protothecosis, algicidal effect, azoles, efinaconazole, luliconazole, ravuconazole

## Abstract

*Prototheca* are unicellular, achlorophyllous, yeast-like microalgae that occur in a wide range of natural habitats. At least five species have been implicated as the causative agents of opportunistic infections of men. Human protothecosis typically manifests as cutaneous, articular, or systemic disease. Treatment is largely empirical with poorly predictable and often unsuccessful outcomes. This is largely due to the frequently observed resistance of *Prototheca* species to conventional antimicrobial agents. This work is the first to perform drug susceptibility profiling exclusively on isolates from human cases of protothecosis. A total of 23 such isolates were tested against amphotericin B and 9 azoles, including efinaconazole and luliconazole, whose activities against *Prototheca* have never been studied before. Efinaconazole was the most active, with median minimum inhibitory concentration (MIC) and minimum algicidal concentration (MAC) values of 0.031 mg/L and 0.063 mg/L, respectively. Fluconazole and luliconazole had the lowest activity, with median MIC and MAC values of 128 mg/L. To conclude, amphotericin B and most of the azoles showed *in vitro* activity, with an algicidal rather than algistatic effect, against *Prototheca*. Still, the activity of individual drugs differed significantly between the species and even between strains of the same species. These differences can be attributed to a species-specific potential for acquiring drug resistance, which, in turn, might be linked to the treatment history of the patient from whom the strain was recovered. The results of this study underscore the potential clinical utility of efinaconazole as a promising therapeutic agent for the treatment of human protothecosis.

## INTRODUCTION

*Prototheca* species are unicellular, achlorophyllous, yeast-like microalgae that occur in a wide range of natural habitats, occupying mostly aquatic niches and living a saprophytic lifestyle (1). These organisms may, under certain conditions, act as opportunistic pathogens to cause a variety of pathologies in both animals and humans. These pathologies are collectively referred to as protothecosis.

Of the 18 currently recognized *Prototheca* species (2, 3, 4), five (P. wickerhamii, P. blaschkeae, P. cutis, P. miyajii, and P. bovis) have been implicated in human protothecosis, with *P. wickerhamii* being responsible for the bulk of the cases (5–7). The disease, which typically manifests in the form of a cutaneous, articular (olecranon bursitis), and systemic infection, remains rare; however, the number of cases has been increasing globally (5). The first case of a *Prototheca* infection in a man was described in Sierra Leone in 1964 (8). Since that time, a total of 211 cases had been identified worldwide by 2017 (9). This number has recently been revised to be 335, with nearly half of the increase being reported only over the last decade (Jagielski, T. et al., paper in progress). The emergence and clinical importance of human protothecosis has been clearly emphasized, with its first reported outbreak occurring in a tertiary care oncology unit in India (10). The problem is further compounded by the fact that there are no standardized therapeutic guidelines for protothecal disease. Treatment is largely empirical, with poorly predictable and often unsuccessful outcomes. This is largely due to the frequently observed resistance of *Prototheca* species to conventional antimicrobial agents (11–13) and to the lack of correlation between the *in vitro* drug susceptibility and the clinical (*in vivo*) response (14). Relatively few studies have investigated the drug sensitivity profiles of *Prototheca* species, with most of the strains being of either animal or environmental origin (11–13, 15–20).

The four main groups of antifungal drugs are polyenes, azoles, allylamines, and echinocandins (21). In the treatment of protothecosis, only the polyene amphotericin B (AMB) and the azoles have been repeatedly used (5). According to the database of the United States Food and Drug Administration (FDA), among the antifungals that have been tested against *Prototheca* species, AMB, fluconazole (FLU), itraconazole (ITZ), ketoconazole (KTZ), miconazole (MCZ), posaconazole (POS), and voriconazole (VRC) can be administered orally or intravenously, whereas efinaconazole (EFZ) and luliconazole (LCZ) can only be used topically (22). The drugs that are addressed in this study are all approved by the FDA for clinical use, except for ravuconazole (RVZ). The use of these drugs, however, tends to be overshadowed by increasing reports on their toxicity and adverse effects. For example, in the case of KTZ, the FDA recommends the discontinuation of the drug due to KTZ-induced renal and hepatic failure (23). Thus, there is consequently an urgent need to develop more effective and less toxic drugs for the treatment of many infections, including protothecal disease.

The purpose of this work was to assess the *in vitro* susceptibility of *Prototheca* species to a panel of 10 antifungal agents that are currently in development or are already available on the pharmaceutical market. This work is the first to focus exclusively on clinical isolates from human cases of protothecosis. Among the drugs tested were AMB and 9 azoles, including EFZ and LCZ, whose activities against *Prototheca* species have never been studied before.

## RESULTS AND DISCUSSION

A total of 3 (13%), 2 (8.7%), and 1 (4.4%) isolates grew in all wells, with the highest MAC concentrations tested being for FLU, ITZ, and LCZ, respectively (Table S1). The remaining 7 drugs showed activity against *Prototheca* isolates at the concentrations employed in this study. For LCZ and FLU, the median MIC and minimum algicidal concentration (MAC) values were equal and amounted to 128 mg/L. LCZ is a relatively new imidazole antifungal agent, and its anti-*Prototheca* activity has not yet been evaluated. Instead, the drug has been found to be effective against a wide spectrum of clinically important fungi, including Aspergillus, Trichophyton, Candida, and Malassezia species (24, 25). The MIC values for the latter three were at least 1,000 times lower than those obtained here for the *Prototheca* species (24).

Likewise, a weak activity toward *Prototheca* was demonstrated for MCZ, the median MIC and MAC values of which were equal to 64 and 128 mg/L, respectively (Table 1; Fig. S1 and S2). In the only study which had previously addressed the activity of MCZ against *Prototheca*, the MIC and MAC values for most of the strains varied greatly from 0.1 to >100 mg/L and from 0.5 to >100 mg/L, respectively (Table 2) (19).

**TABLE 1:** Minimum inhibitory concentrations (MICs) and minimum algicidal concentrations (MACs) of drugs tested for 23 *Prototheca* species isolates^*a*^

Species^*b*^	Minimum inhibitory concentration (MIC) / minimum algicidal concentration (MAC) [mg/L]^*c*^									
	AMB	EFZ	FLU	ITZ	KTZ	LCZ	MCZ	POS	VRC	RVZ
*P. blaschkeae* (*n *= 1)	0.5/0.5	0.031/0.063	256/256	2/2	1/4	64/128	64/256	2/2	8/8	0.063/0.125
*P. bovis* (*n *= 3)	0.5 to 1/0.5 to 1^R^	0.125 to 0.5/0.125 to 0.5^R^	64 to 128/64 to 128^R^	4 to >32/4 to >32^R^	4 to 32/4 to 32^R^	64 to 256/64 to 256^R^	32 to 128/32 to 128^R^	1 to 32/1 to 32^R^	4 to 16/8 to 16^R^	0.5 to 1/1^R^
	0.5/0.5^M^	0.25/0.25^M^	128/128^M^	32/32^M^	8/8^M^	128/256^M^	128/128^M^	4/4^M^	8/8^M^	0.5/1^M^
*P. ciferrii* (*n *= 2)	0.5; 1/0.5; 1	0.008; 0.063/0.008; 0.063	128/128	1; 2/1; 2	1; 2/2	128/128	64; 128/64; 128	1; 2/1; 2	2/2	0.125/0.125
*P. cutis* (*n *= 1)	0.5/1	0.063/0.063	64/128	4/16	4/4	128/128	64/64	8/8	4/8	0.125/0.125
*P. miyajii* (*n *= 2)	0.25; 0.5/1	0.016; 0.063/0.031; 0.5	128/>256	4; 32/16; 64	4/4	16; 64/16; 128	64/128	32/128	4; 8/4; 16	0.125/0.25; 0.5
*P. moriformis* (*n *= 1)	0.5/1	0.016/0.031	128/>256	8/32	8/16	128/>256	32/128	128/256	2/2	32/32
*P. pringsheimii* (*n *= 1)	1/1	0.063/0.063	128/128	4/4	1/2	128/128	64/128	2/2	2/2	0.125/0.5
*P. wickerhamii* (*n *= 12)	0.125 to 1/ 0.125 to 1^R^	0.008 to 0.063/0.008 to 0.125^R^	32 to 256/32 to 256^R^	2 to >32/2 to >32^R^	1 to 8/1 to 8^R^	32 to 128/32 to 256^R^	8 to 128/8 to 128^R^	0.5 to 8/0.5 to 8^R^	1 to 8/1 to 8^R^	0.031 to 0.25/0.031 to 0.25^R^
	0.5/0.5^M^	0.031/0.031^M^	128/128^M^	4/4^M^	2/2^M^	128/128^M^	64/64^M^	3/3^M^	4/4^M^	0.063/0.063^M^
Total (*n* = 23)	0.125 to 1/0.125 to 1^R^	0.008 to 0.5/0.008 to 0.5^R^	32 to 256/32 to >256^R^	1 to >32/1 to >32^R^	1 to 32/1 to 32^R^	16 to 256/16 to >256^R^	8 to 128/8 to 256^R^	0.5 to 128/0.5 to 256^R^	1 to 16/1 to 16^R^	0.031 to 32/0.031 to 32^R^
	0.5/0.5^M^	0.031/0.063^M^	128/128^M^	4/4^M^	2/2^M^	128/128^M^	64/128^M^	4/4^M^	4/4^M^	0.125/0.125^M^

a AMB, amphotericin B; EFZ, efinaconazole; FLU, fluconazole; ITZ, itraconazole; KTZ, ketoconazole; LCZ, luliconazole; MCZ, miconazole; POS, posaconazole; VRC, voriconazole; RVZ, ravuconazole.

b The study included strains provided by collaborating researchers (*n* = 8) and purchased from culture collections, as detailed in Supplementary Table 1: ATCC, American Type Culture Collection (*n* = 4), USA; CBS, CBS-KNAW Culture Collection, Westerdijk Fungal Biodiversity Institute, The Netherlands (*n* = 2); Dmic, Fungal Culture Collection, Mycology Department, National Institute of Infectious Diseases, Argentina (*n* = 1); IFM, Medical Mycology Research Center, Chiba University, Japan (*n* = 3); IHEM, Institute of Hygiene and Epidemiology-Mycology Laboratory, Belgium (*n* = 2); SAG, The Culture Collection of Algae, Göttingen University, Germany (*n* = 3).

c Ranges (R) and medians (M) were given only for drugs tested against *P. bovis*, *P. wickerhamii*, and all *Prototheca* species combined. They are indicated by the superscripts (R and M, respectively) of the values that are entered in Table 1.

**TABLE 2:** Comparison of the minimum inhibitory concentration (MIC) and minimum algicidal concentration (MAC) ranges of tested drugs for *Prototheca* species strains, as assessed in the present and past studies^*a*^

Drug^*b*^	MIC/MAC, range [mg/L]^*c*^				Reference
	This study	Past studies			
		Clinical strains	Environmental strains	Total	
AMB	0.125 to 1/0.125 to 1	0.15 to 12.5/ND	ND	0.064 to 12.5/0.19 to 25	6
		0.25 to 4/ND	0.064 to 0.5/ND		11
		0.094 to 3/ND	ND		12
		0.125 to 1***/ND	ND		15
		0.25−0.5*/ND	ND		17
		0.19 to 3.12/0.38 to 25	0.09 to 0.75/0.19−12.5		19
		0.244 to 0.976/ND	ND		26
		ND	0.03 to 4**/ND		27
EFZ	0.008 to 0.5/0.008 to 0.5	ND	ND	ND	ND
FLU	32−256/32 to >256	8 to >200/ND	ND	8 to >256/ND	6
		>256/ND	ND		12
		>128*/ND	ND		17
		ND	64 to 256**/ND		27
ITZ	1 to >32/1 to >32	0.39 to >100/ND	ND	0.12 to >100/ND	6
		>32/ND	2 to >32/ND		11
		1 to >32/ND	ND		12
		4 to >32/ND	0.25 to >32**/ND		16
		0.12−1*/ND	ND		17
		ND	0.5 to 16**/ND		27
KTZ	1 to 32/1 to 32	1 to 60/ND	ND	0.25 to 60/ND	6
		0.25 to >32/ND	ND		12
		ND	8 to 32**/ND		27
LCZ	16 to 256/16 to >256	ND	ND	ND	ND
MCZ	8−128/8 to 256	0.1 to >100/0.5 to >100	1 to >100/5 to >100	0.1 to >100/0.5 to >100	19
POS	0.5−128/0.5 to 256	0.38 to >32/ND	ND	0.03 to >32/ND	12
		0.03 to >32/ND	0.03 to 0.25/ND		16
		0.12−1*/ND	ND		17
VRC	1 to 16/1 to 16	0.15 to >16/ND	ND	0.25 to >32/ND	6
		0.25 to >32/ND	ND		12
		0.25 to >32/ND	0.25 to 4/ND		16
		0.5 to 32*/ND	ND		17
RVZ	0.031 to 32/0.031 to 32	0.03 to 0.25/ND	0.03/ND	0.03 to 0.25/ND	16

a Superscripts of *, **, and ***, next to selected values indicate that the ranges refer to strains of either clinical or environmental origin (not yet having been specified for individual strains). That is, there are 20 clinical (bovine) strains and 6 environmental strains (*), strains whose majority were isolated from the environment (**), and strains whose majority were isolated from bovine mastitis cases (***).

b AMB, amphotericin B; EFZ, efinaconazole; FLU, fluconazole; ITZ, itraconazole; KTZ, ketoconazole; LCZ, luliconazole; MCZ, miconazole; POS, posaconazole; VRC, voriconazole; RVZ, ravuconazole.

c MIC, minimum inhibitory concentration; MAC, minimum algicidal concentration; clinical strains, strains isolated from cases of human and animal protothecosis; environmental strains, strains isolated from various environmental sources; ND, not determined. The ranges that are based on values that were calculated for human strains are underlined.

The four other azoles were shown to be much more effective, with their median MICs ranging from 2 mg/L (KTZ) to 4 mg/L (ITZ, POS, VRC). For all of these drugs, the MICs overlapped with the MACs, suggesting their algicidal effect (Table 1; Fig. S1 and S2). The median MICs of ITZ, VRC, and POS were all 4 mg/L, being 4 times lower, two times higher, and 16 times higher than the respective values (of the same drugs) that were assessed in a previous study that was performed on strains isolated from cases of bovine mastitis or from the environment (16). Even higher were the MICs of the three azoles (ITZ, VRC, POS) from this study, compared with the MICs of these drugs calculated for *P. bovis* strains that were retrieved from mastitis cows only (17).

The third most potent anti-protothecal drug was AMB, with its median MIC and MAC values being 0.5 mg/L (Table 1; Fig. S1 and S2). This is in agreement with previously reported susceptibility results for this drug, the median MICs of which typically ranged from 0.064 mg/L to 12.5 mg/L (Table 2) (6, 11, 12, 15, 17, 19, 26, 27). AMB is currently among the front-line drugs for the treatment of human protothecosis. It is a drug of first choice for *Prototheca* systemic infections. The overall success rate for AMB treatment has been calculated at 77%, compared to 67%, 66%, and 55% for treatment with FLU, ITZ, and KTZ, respectively (5). Still, an important disadvantage of using AMB is its high cytotoxicity, which may produce serious kidney and liver failure (28).

The drug with the second highest activity against *Prototheca* species was RVZ, the median MIC and MAC values of which were both assessed at 0.125 mg/L (Table 1; Fig. S1 and S2). So far, only one study has reported on the activity of RVZ against *Prototheca* species. The median MIC value was 4 times lower than that observed in the present study (0.03 versus 0.125 mg/L), with the difference potentially being explained by the species affiliation and origin of the analyzed strains (16). The previous study chiefly involved *P. bovis* and *P. ciferrii* strains that were isolated from animal and environmental sources. The seemingly broad range of the RVZ MICs was due to a single strain of P. moriformis, for which the MIC and MAC were as high as 32 mg/L (Table 1; Fig. S1 and S2; Table S1).

The highest anti-*Prototheca* activity was shown for EFZ, a novel compound of the triazole series (median MIC/MAC, 0.031/0.063 mg/L; range, 0.008 to 0.5 mg/L for both MIC and MAC) (Table 1; Fig. S1 and S2). To the best of the authors’ knowledge, this study is the first to investigate the *in vitro* susceptibility of *Prototheca* species to EFZ. The drug has been demonstrated to be highly active against a variety of pathogenic fungi, including dermatophytes and *Candida* species (29–31). For instance, the EFZ MICs for Trichophyton mentagrophytes and Trichophyton rubrum usually did not exceed 0.015 mg/L, whereas for C. albicans, these values were even lower, typically below 0.004 mg/L (30, 31). Owing to a broad spectrum of antifungal activity, EFZ was approved by the FDA in 2014 for the topical treatment of superficial infections and for onychomycosis, in particular (32). The clinical use of EFZ has been advocated due to its low toxicity being demonstrated in animal studies and its limited adverse events on clinical trials (33–35).

The MAC values were equivalent to the corresponding MIC values for all of the drugs, except for EFZ and MCZ. The latter two had MAC values that were two times higher than their MIC values (Table 1; Fig. S1 and S2). Thus, the analyzed drugs seem to exert algicidal, rather than algistatic, effects on *Prototheca*. Although most of the species were represented by single strains, some differences between their mean MIC and MAC values were observed. For example, *P. moriformis* had its MIC values for RVZ and POS at least 32 and 4 times higher, respectively, than did any other *Prototheca* species. Finally, the MICs of EFZ for *P. bovis* were clearly higher, at least by twofold, compared to other *Prototheca* species.

The interspecies differences in drug susceptibility have been described previously. Studies performed on strains from mastitic cows showed AMB and azoles (ITZ, KTZ, POS, VOZ) to be less active against *P. bovis* than against P. blaschkeae (12, 15). The lower activity of AMB and ITZ toward *P. bovis* was observed compared with that toward P. ciferrii (11). Also, in this work, strains of *P. ciferrii* were conspicuously more susceptible to all but three of the azoles that were tested (i.e., EFZ, ITZ, KTZ, POS, VRC, RVZ), compared with *P. bovis* (Table 1; Fig. S1 and S2).

## MATERIALS AND METHODS

A total of 23 *Prototheca* species isolates were used in this study. They were all originally collected from human clinical samples and represented cases of symptomatic protothecal disease. Of these, the majority were systemic infections (*n* = 9; 39.1%), and these were followed by skin/nail lesions (*n* = 7; 30.5%), enteric infections (*n* = 3; 13%), neuroinfections (*n* = 2; 8.7%), skeletal lesions (*n* = 1; 4.4%) and synovial fluid (*n* = 1; 4.4%) (Table S1). Pichia kudriavzevii ATCC 6258 was used as a quality control for the drug susceptibility testing.

The strains were provided, on commercial terms, from international culture collections or were donated by collaborating researchers (Table S1). The strains were all cryopreserved in Viabank Bacterial Storage Beads (MWE Medical Wire, United Kingdom) at −70°C. The strains were revived by streaking a loopful (10 μL) of the frozen culture onto Sabouraud Dextrose Agar (SDA) (Biomaxima, Poland) plates, and they were subsequently incubated under aerobic conditions at 30°C for 72 h.

For all strains, species-level identification was performed, either in this study or elsewhere (3), via a polymerase chain reaction-restriction fragments length polymorphism (PCR-RFLP) analysis of the partial *CYTB* gene as a typing tool (36). To avoid misidentifications of *P. bovis* as *P. ciferrii*, strains that were identified as *P. bovis* had their *CYTB* PCR products sequenced and analyzed via the Prototheca-ID web application (37).

In the absence of universally accepted guidelines that were specifically applicable to *Prototheca* species, the determination of the MIC was performed using the broth microdilution method in 96-well microtiter plates, strictly following the Clinical and Laboratory Standards Institute (CLSI) protocol (M27-A3) for the drug susceptibility testing of yeasts (38). The MIC and MAC values were determined in RPMI 1640 medium (Sigma-Aldrich, Poland) with incubation at 30°C for 72 h. The only modification of the CLSI protocol was that the suspension of the protothecal inoculum was adjusted to a 6 McFarland turbidity standard to provide the CLSI recommended inoculum size (i.e., 1 to 5 × 10^3^ CFU/mL) (39).

A total of 10 drugs were tested, including AMB, EFZ, FLU, ITZ, KTZ, LCZ, MCZ, POS, VRC, and RVZ, which were supplied by Sigma-Aldrich, Poland. Working solutions were prepared in dimethyl sulfoxide (Sigma-Aldrich, Poland) immediately before use.

Drugs were tested at doubling concentrations (i.e., from 0.016 to 2 mg/L [AMB], 0.004 to 2 mg/L [EFZ], 2 to 256 mg/L [FLU, LCZ, MCZ], 0.25 to 32 mg/L [ITZ, KTZ, VRC], 0.25 to 256 mg/L [POS], and 0.016 to 32 mg/L [RVZ]). The MIC was described as the lowest concentration of the tested compound that completely inhibited the algal growth, as observed with the naked eye. The MAC values were determined essentially as was described previously (15). Briefly, 100 μL aliquots from the control wells and wells corresponding to the MICs, twofold, and fourfold were spread onto SDA plates. After 72 h of incubation at 30°C, the number of colonies per plate was counted. The MAC was defined as the lowest drug concentration that killed at least 99.9% of the algal cells, compared to the control. All of the assays for each strain were performed in triplicate. Only if 2 replications showed identical results were final MIC or MAC values assigned.

### Conclusions.

To conclude, the results of this study indicate that AMB and most of the azoles are active against *Prototheca* clinical isolates and mostly exert an algicidal effect. Still, the activities of individual drugs differ significantly between species and between strains of the same species. Apart from a small number of strains representing different species, which marks an important limitation of the study, these differences can be explained by a species-specific potential for the acquisition of drug resistance, which, in turn, might be linked to the treatment history of the individual patient from whom the strain was recovered. Finally, the differences in the efficacies of certain azoles between this study and previous studies may relate to the strain origin (source of isolation) and, as far as the protothecal disease is concerned, may reflect the biological and clinical peculiarities of the infections of the human and animal hosts. These peculiarities may have a chance to be disclosed with the advent of advanced genomic approaches, which have recently been introduced into the field of *Prototheca* research (39, 40). The genome-wide analytical tools are also believed to reveal molecular determinants of drug metabolism and resistance, which could further be translated into the development of new therapeutics against *Prototheca*. In parallel, new preparations continue to be tested for their anti-*Prototheca* activity. Recent studies have shown various chemical substances as new, promising options for the treatment of protothecosis, including iodine-containing carbamates (15), antimicrobial peptides (41), and silver nanoparticles (42, 43). In this study, EFZ, having shown the highest activity against the *Prototheca* algae, should be considered to be a potential therapeutic alternative for human protothecosis.
