# Supplementary material for: Drug Susceptibility Profiling of Prototheca Species Isolated from Cases of Human Protothecosis
Source: Antimicrob Agents Chemother. 2023 Mar 21;67(4):e01627-22. doi: 10.1128/aac.01627-22 (PMC10112244; doi:10.1128/aac.01627-22)
Supplement: Supplemental file 2 — Supplemental material. Download aac.01627-22-s0002.pdf, PDF file, 0.3 MB [file aac.01627-22-s0002.pdf]

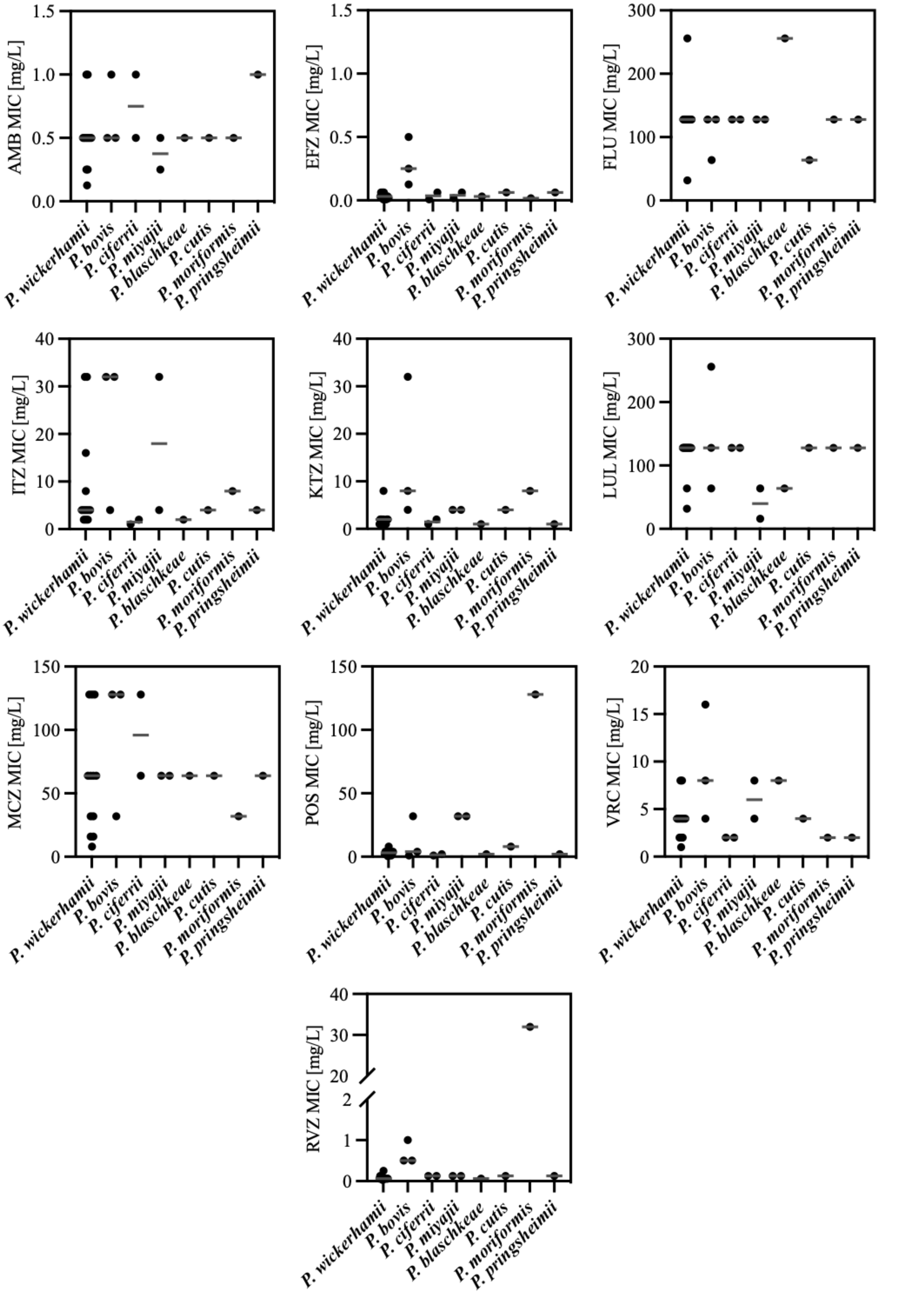

Supplementary Figure 1. Minimum inhibitory concertations (MICs) of drugs tested for 23 *Prototheca* sp. isolates.

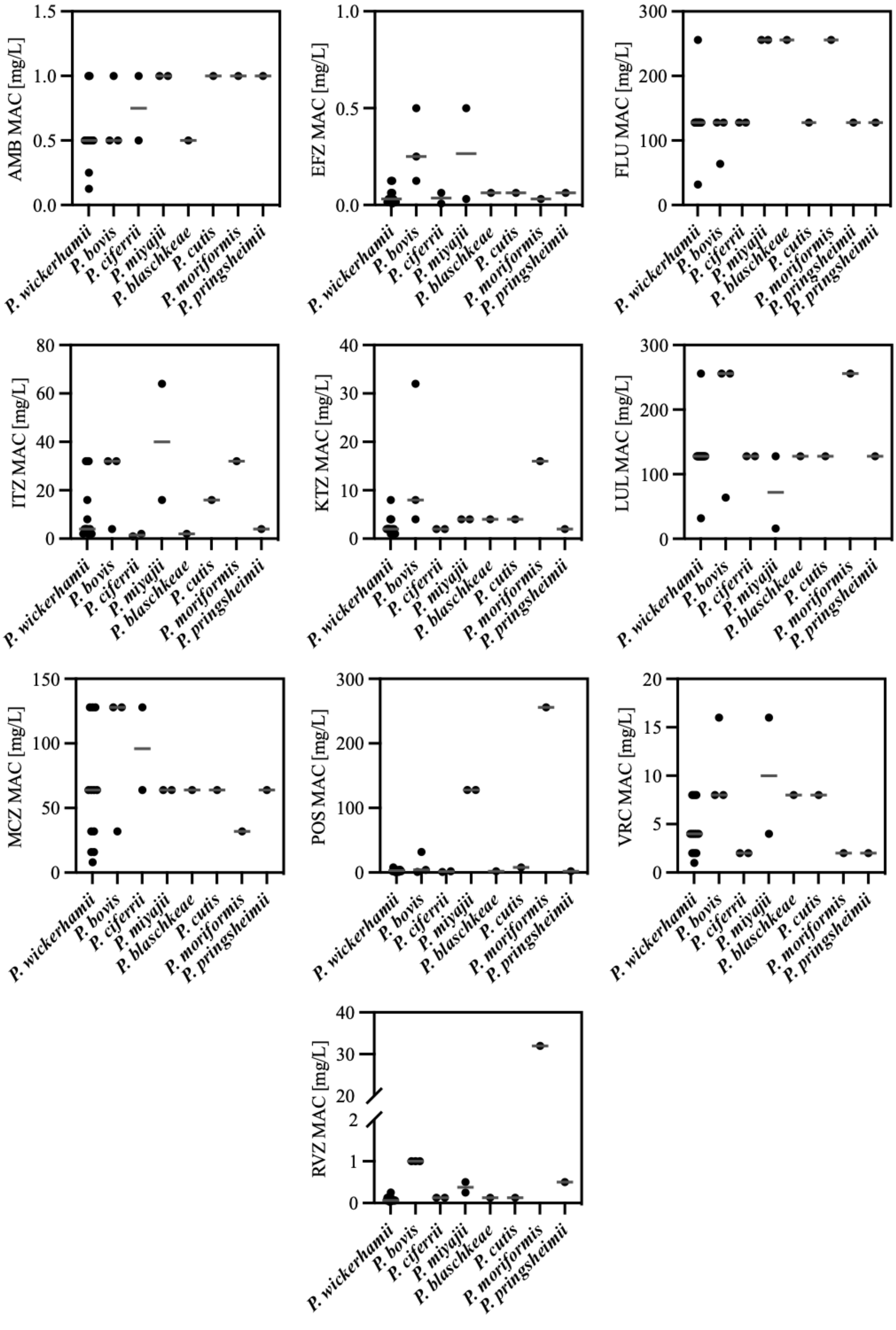

Supplementary Figure 2. Minimum algicidal concentrations (MACs) of drugs tested for 23 *Prototheca* sp. isolates.
